# Attention capture in birds performing an auditory streaming task

**DOI:** 10.1371/journal.pone.0235420

**Published:** 2020-06-26

**Authors:** Huaizhen Cai, Micheal L. Dent

**Affiliations:** Department of Psychology, University at Buffalo, The State University of New York, Buffalo, New York, United States of America; Universidad de Chile, CHILE

## Abstract

Numerous animal models have been used to investigate the neural mechanisms of auditory processing in complex acoustic environments, but it is unclear whether an animal’s auditory attention is functionally similar to a human’s in processing competing auditory scenes. Here we investigated the effects of attention capture in birds performing an objective auditory streaming paradigm. The classical ABAB… patterned pure tone sequences were modified and used for the task. We trained the birds to selectively attend to a target stream and only respond to the deviant appearing in the target stream, even though their attention may be captured by a deviant in the background stream. When no deviant appeared in the background stream, the birds experience the buildup of streaming process in a qualitatively similar way as they did in a subjective paradigm. Although the birds were trained to selectively attend to the target stream, they failed to avoid the involuntary attention switch caused by the background deviant, especially when the background deviant was sequentially unpredictable. Their global performance deteriorated more with increasingly salient background deviants, where the buildup process was reset by the background distractor. Moreover, sequential predictability of the background deviant facilitated the recovery of the buildup process after attention capture. This is the first study that addresses the perceptual consequences of the joint effects of top-down and bottom-up attention in behaving animals.

## Introduction

To understand the sounds surrounding us, humans and other animal species have evolved the mechanisms to disentangle sound mixtures arriving at the auditory system into separate streams. However, at the same time, auditory distractors are ubiquitous in daily acoustic scenes, which affects a listener’s attentional set and the sound segregation process. Hence, it is essential for living organisms to evolve the capability to handle the sound distractors when disentangling complex auditory scenes, while simultaneously maintaining some vigilance about the unexpected and potential danger revealed by the sound distractors in the environment [1].

The auditory streaming process has been investigated in both humans [2–4] and other animal species [5–9]. Auditory streaming involves segregating simultaneous sound streams emitted by different sources and grouping sequential streams emitted by the same sound source, depending on the spectral and temporal cues embedded in these sound streams [10, 11]. As simplified substitutes for natural complex sound scenes, ABA-ABA-…or ABAB… patterned pure tone sequences have been used across studies to investigate the physical properties and neural mechanisms of the auditory streaming process. By changing the acoustic characteristics of the A and B tones (such as frequency, temporal envelope, phase spectrums, and so on), listeners can either hear a coherent stream of alternating A and B tones, or two separate streams, one that consists of A tones and the other that consists of B tones [4].

In humans, listeners’ attentional sets have been unequivocally demonstrated to affect the auditory streaming process [11–20]. Neglect patients with asymmetric attentional deficits tend to show less streaming perception for sounds presented to the deficit side than those presented to the normal side [20]. A dynamic focus of attention to different acoustic features of a target can enhance the binding of target features over time [11, 12] and the segregation of a target from backgrounds [11, 13]. Congruent results have also been obtained in neurophysiological studies on the effects of selective attention on the segregation of the target stream in competing sound scenes. Top-down selective attention can enhance the amplitude of neural activity to attended stimuli [14, 15], modulate cortical plasticity in the direction of facilitating the segregation of attended stimuli [16], synchronize global neural oscillations to the spectrotemporal features of the attended stimuli [17, 18], and modulate noise correlations of cortical neurons that are activated by the attended stimuli, which enhances the neural representation of the attended stimuli [19].

Accumulated studies have also demonstrated a buildup effect in auditory streaming processes [2, 5, 6, 21–27], where presenting pure tone sequences to listeners for longer periods of time is more likely to generate a segregated percept [5, 7, 24]. The buildup process is accelerated for larger acoustical differences between the A and B tones, faster repetition rates of the sequence, or more reliable sound continuity within each stream [24, 28–31]. It has been addressed in several animal species that tonotopically overlapped neurons in the ascending auditory pathway tend to adapt to different tones in the pure tone sound sequence selectively over time [22, 24, 29, 32], which both qualitatively and quantitatively accounts for the psychophysical observations of the buildup process [5, 33, 34]. Also, the endogenous stream-brain wave dynamically phase locking to the temporal structure of the sound target over time can be modified to affect the buildup process [35].

Similar to the auditory streaming process, a listener’s attentional set also matters in the build-up process. For example, attending to a competing sound presented to one ear reduces the build-up process of an ABA-… sequence presented to the unattended contralateral ear [20]. A short switch of attention away from the attended sound to another sound [36] or visual targets [37] can deteriorate the buildup process. An abrupt change of location, intensity, frequency, and tempo in the sound can reset the buildup process [31, 33]. Additionally, buildup-related neural activity was reduced when listeners ignored the ABA- patterned sound stimuli, and this buildup-related neural activity showed a right-hemisphere dominance [38]. Top-down attention can facilitate the refinement of brain wave phase locking to the attended sound, which accelerates the buildup process [39, 40]. Nevertheless, the buildup process is not completely under voluntary attentional control; listeners cannot intentionally avoid the buildup process even though avoiding it can facilitate the task performance [27].

It is clear that both top-down selective attention and bottom-up stimulus-driven attention can affect the auditory streaming process. Studies on humans have indicated that sudden changes in unattended stimuli may elicit an involuntary attention switch to the unattended stimuli [41–43], also called attention capture. The effect of attention capture has been interpreted as the vigilance of the central nervous system to detect inconsistencies in stimuli [44], and attention capture is subject to the top-down process [45]. The prefrontal cortex is strongly involved in the involuntary attention orientation process, as deterioration of the frontal cortex is associated with a larger attention capture effect [46–49]. In the auditory modality, top-down controlled and bottom-up triggered attention seem to activate largely the same cortical networks [50], while in vision, accumulated evidence from neglect patients suggests segregated cortical areas activated for top-down controlled and bottom-up triggered attention [1].

The effect of top-down processing on attention capture in the auditory domain has been widely studied in humans by recording the event-related brain potentials (ERPs) [51, 52]. Conflicting results were obtained about the effects of the top-down processing on the deviant-elicited ERPs [53–57], which implies that the interaction between top-down and bottom-up attentional processes may vary depending on context [1, 58]. Nevertheless, consistent behavioral results were obtained in these studies, where attention capture generally leads to prolonged response times and declined performance in the primary task [53–55, 57]. Nevertheless, [45] has proposed that in vision, increasing perceptual load (e.g., number of items needed to be perceived) in a primary task typically eliminates the distractor effect, while increasing the cognitive control load (e.g., working memory) increases the distractor interference, especially when selective attention is involved in the primary task [53, 59].

Furthermore, in auditory perception, the effects of attention capture also vary depending on the characteristics of deviants, the temporal relationship of the deviants with the target, and other top-down processes in addition to selective attention [41]. More salient deviants tend to capture attention more than less salient deviants, therefore deteriorating the listener’s performance in the primary task more than the less salient deviants [60, 61]. The distractive effect decreases as the onset-to-onset interval between the distractor and the target increase [49], which is accompanied by an earlier and stronger N1 ERP response elicited by the target [57]. Studies in humans have indicated that the distractive effect disappears as the onset-to-onset interval between the deviant and the target became longer than 560 ms [49, 61]. Moreover, when the deviants convey some information about the forthcoming targets or when the deviants have predictable attributes and occurrences, the deterioration effect of attention capture is reduced [62–64]. In auditory scene analysis, knowledge of sound regularity and predictability helps to orient the cognitive resources (such as attention) for the future sound stimulus in advance, which facilitates the subsequent segregation of sound targets. In a dichotic listening task, task-irrelevant sound stimuli with repeated frequency components tended to be less distractive than task-irrelevant stimuli with random frequency components [65]. Nevertheless, when the deviants appear right before or at the same time as the target, the deviants promote the performance in the primary task. This short-lived facilitation effect may be caused by a higher arousal level elicited by deviants [57], or the transiently narrowed attentional spotlight, where neurons transiently bias to the shared acoustic features between the deviants and targets [66].

The aforementioned studies of attention capture were mostly conducted in human subjects where listeners were instructed to ignore the distractors when performing the primary task. There have been limited studies systematically addressing the attention capture process in animals [67–69], although animal models have been widely used to investigate the neural mechanisms of auditory attention in the auditory streaming process [18, 70–72]. It is unknown whether attention capture affects animals’ perception of auditory streaming in the same way as in humans. Also, in animals, it is unknown if the interaction mechanism of goal-directed and stimulus-driven attention in the auditory streaming process is functionally similar to that in humans. Birds have been widely used as comparative animal models to unveil the neurobiological mechanisms underlying auditory perception [73–75]. Also, a plethora of psychophysics experiments have been conducted on budgerigars to understand complex sound perception in this species [76–81]. Moreover, a previous study showed that birds experienced auditory streaming and the buildup effect in a qualitatively similar way as that in humans [5]. Hence, the present study used budgerigars to explore how this species experiences attention capture in an objective auditory streaming task and determine how the predictability of the attention capturer will affect the auditory streaming process in behaving birds.

## Materials and methods

### Ethics statement

All procedures were approved by the University at Buffalo, SUNY’s Institutional Animal Care and Use Committee [IACUC] and were in accordance with *the Guide for Care and Use of Laboratory Animals*.

### Animal subjects and housing

Seven adult budgerigars (5 males and 2 females) were used as subjects. Birds were either purchased from local pet suppliers or bred in the vivarium. Birds were individually housed and had free access to water. The vivarium was kept on a 12 hour day/night cycle at the University at Buffalo, SUNY. Birds were maintained at 90–95% of their free-feeding body weights for the duration of the experiment. The birds were tested in two daily sessions, with each session lasting 45–60 min, 5–7 days a week. The birds typically finished about 100–300 trials in a session.

### Stimuli

All acoustic stimuli were repeated sinusoidal phase tones with a sample rate of 40 kHz and 16-bit resolution. Unless otherwise mentioned, all sound stimuli were generated digitally in MATLAB and delivered at 90 dB SPL as measured by a Larson-Davis sound level meter (Model 825) placed at the location of the bird’s head in the wire cage. The tone durations were consistent at 80 ms in all conditions, with 10-ms linear amplitude onset and offset ramps. The inter-tone intervals were 80 ms in the target and the background streams. The tone frequencies used in the sound stimuli will be addressed in detail in the next section.

### Behavioral apparatus and procedure

**Fig 1:**
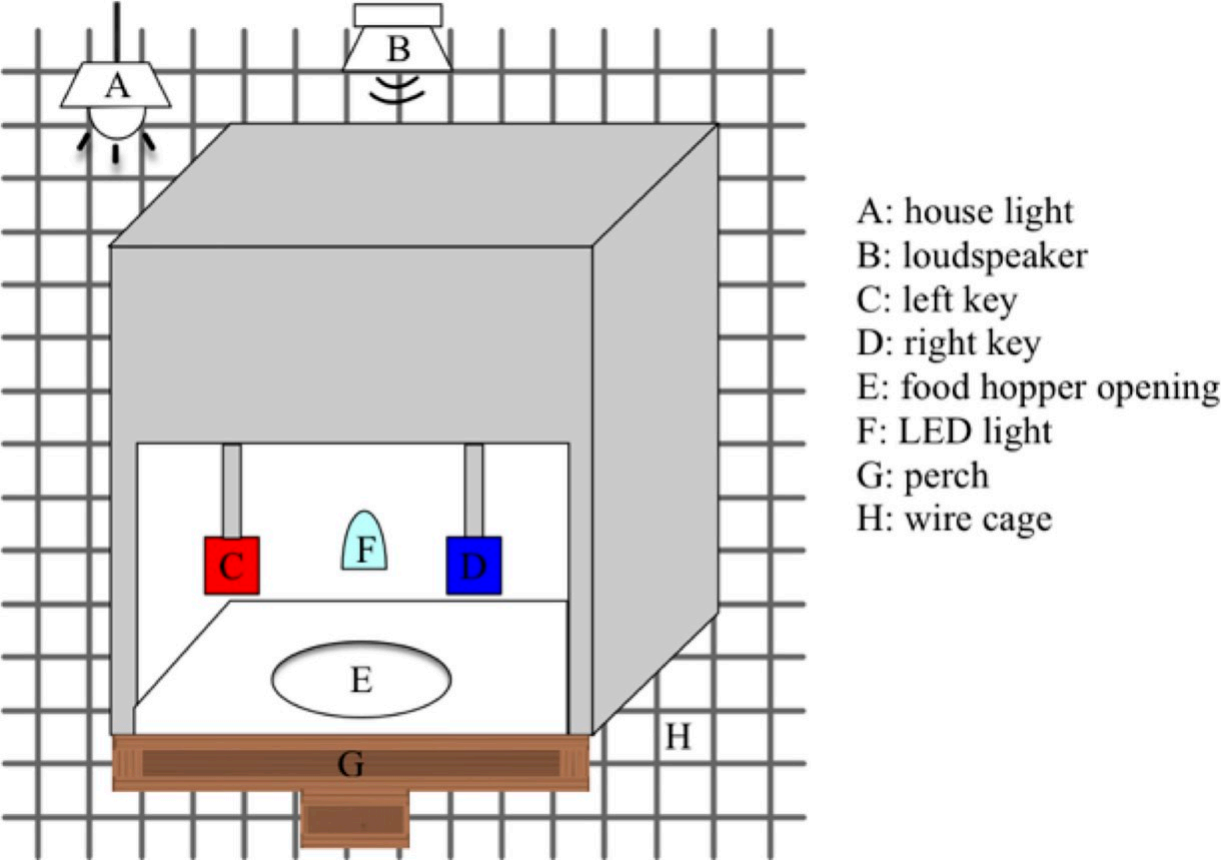
Schematic of the experimental apparatus. The food hopper pops up to allow access to millet through the opening. The LED light is turned on as a secondary reinforcer.

The birds were trained in an objective auditory streaming paradigm using operant conditioning procedures. The experimental setups have been described in [5]. The birds were trained to initiate a trial and the stimulus presentation by pecking the left key (Fig 1). Following the presentation of the stimulus, the birds were trained to peck the right key when discriminating a frequency deviant inserted in the target stream.

In each session, 70% of trials were testing trials, where a frequency deviant sequentially randomly appeared in the target stream; 30% of trials were sham trials, where the target stream exclusively consisted of standard tones. For testing trials, responding to the frequency deviant within 800 ms after the onset of the deviant was counted as a ‘hit’, which then terminated the stimulus presentation immediately and the birds were reinforced by 1.2–1.5 s access to millet. Any key pecks before the onset of the frequency deviant led to immediate termination of the sound stimulus and the birds were punished with 2–6 s blackout of the house light. The stimulus was replayed in the next trial. No responses during the response window were counted as ‘misses’. The animals were neither punished nor reinforced after miss trials. For sham trials, the response window started simultaneously with the stimulus and ended 240 ms after the stimulus offset, which matched the latest possible response window offset of testing stimuli. This was designed to prevent the birds from responding immediately after the stimulus offset to obtain some chances of millet reward while 100% avoiding blackout. Key pecks within the response window for sham trials were counted as ‘false alarms’. False alarm responses led to immediate termination of the sound and the birds were punished with 2–6 s blackout of the house light. No responses during the presentation of sham stimuli were counted as ‘correct rejections’, and were followed by a 30%-80% probability of millet reinforcements.

**Fig 2:**
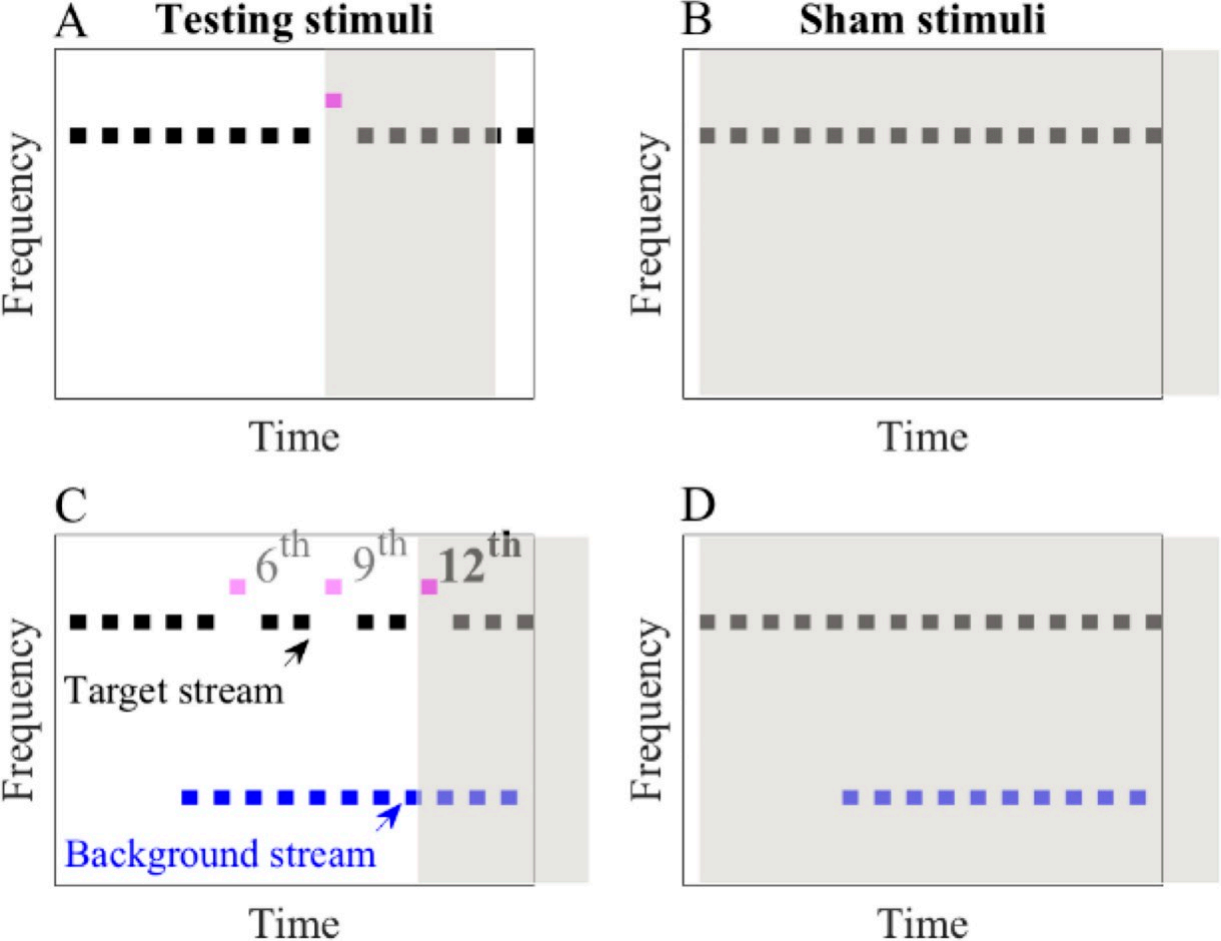
Stimuli used in the training and baseline experiment. The horizontal axis is time; the vertical axis is frequency. The grey areas indicate the response windows. Blue and black streams represent background and target streams, respectively. A & B: testing stimuli and sham stimuli used in training. C & D: testing stimuli and sham stimuli with the flat background stream (1890 Hz tones) used in the baseline experiment. The frequency deviant in the target stream randomly appeared at the 6^th^, 9^th^, or the 12^th^ tone in C (pink dashes), and could be 0.5%, 2%, 4%, 10% (15%) higher than the frequency of the target standard tones (3000 Hz).

In the beginning, all birds were trained to discriminate a relatively salient frequency deviant (10% or 15% higher than the standard tones of 3000 Hz) randomly inserted in a short (4 tones to begin) pure tone sequence (Fig 2A), and to withhold their responses when the sequence exclusively consisted of standard tones (Fig 2B). Once the birds reached the criterion of higher than 80% hit rate and lower than 20% false alarm rate, the time course of the stimuli was extended across sessions (2 more tones were added) until the stimuli consisted of 15 repetitions of the A- patterned pure tone sequence, as shown in Fig 2A. Once a bird could perform the discrimination task with pure tone sequences consisting of 15 tones, a background stream was gradually introduced. The intensity of the background stream started at 40 dB SPL and gradually increased to 90 dB SPL (i.e., the same intensity as the target stream) across training sessions. The background stream was temporally interleaved with the target stream, and was delayed by 4 tones relative to the target stream as a prime cue for the bird’s auditory attention (as shown in Fig 2C & 2D). The tone frequency of the background stream was consistent at 1890 Hz, which was 8 semitones (STs) lower than that of the target stream (which was 3000 Hz). The frequency combination of target and background standard tones was the same in all subsequent conditions. Once the bird performed the discrimination task in the presence of a background stream, baseline data collection started, followed by condition 1 and then condition 2. To calculate thresholds, multiple target frequency deviants were used across trials in each condition, which took the values of 0.5%, 2%, 4%, and 10%/15% higher than that of the target standard tones (depending on the performance of the bird, the number of the most salient frequency deviants in a block was adjusted, and was either 10% or 15% to maintain motivation levels). The target frequency deviant randomly appeared at the 6^th^, 9^th^, or the 12^th^ tone in the target stream.

**Fig 3:**
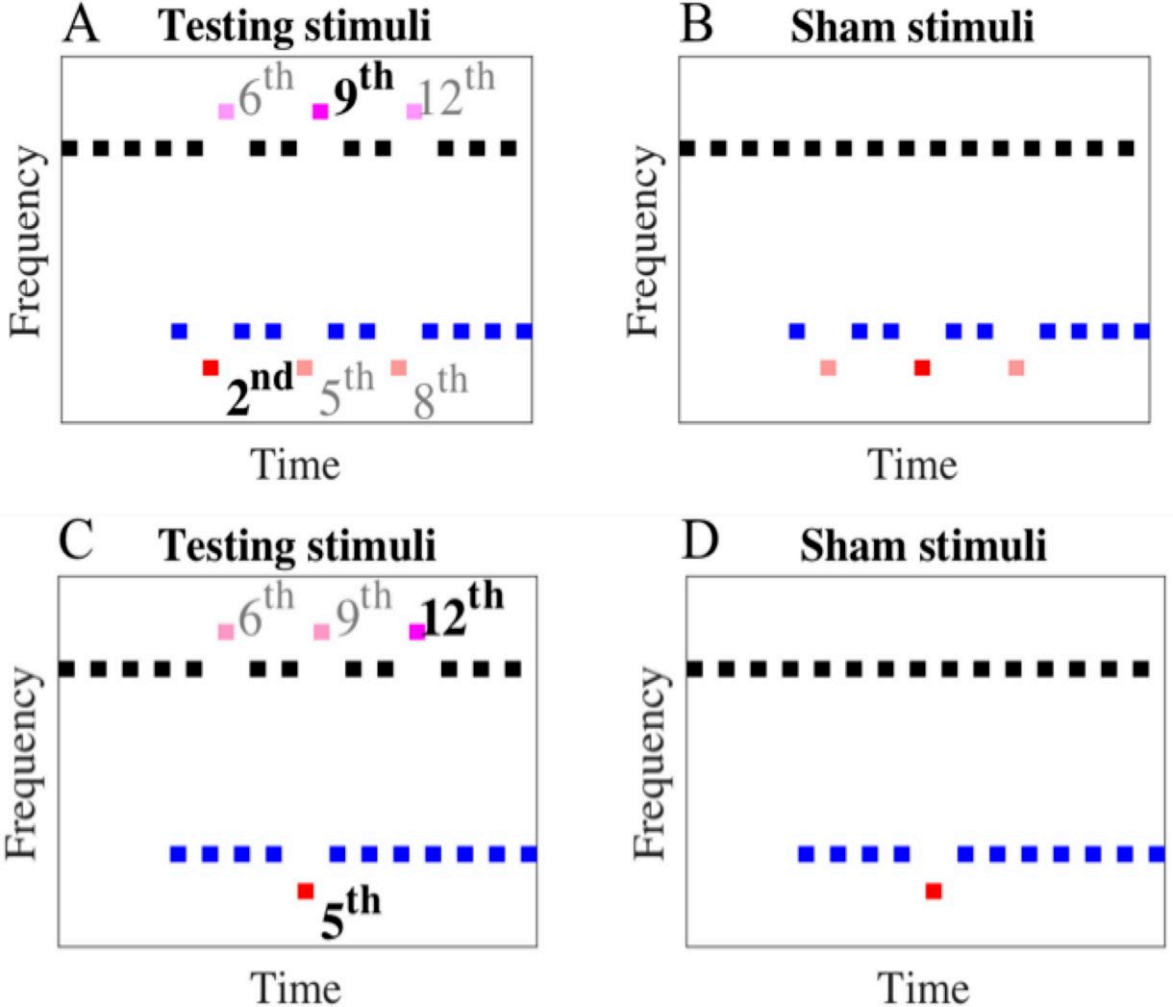
Testing and sham stimuli used in condition 1 (A & B) and condition 2 (C & D). The target deviant (pink dash), target, and background standard tones were the same as Fig 2C. The background deviant (red dash) randomly appeared at one of the three sequential positions (2^nd^, 5^th^, or the 8^th^ tone) in condition 1 while consistently at the 5^th^ tone in condition 2.

After completing the baseline condition, background deviants were introduced and the birds completed condition 1 and then condition 2. In condition 1, the background deviants appeared unpredictably at one of three sequential positions (at the 2^nd^, 5^th^, or the 8^th^ background tone) in the background stream of both the testing and sham trials. The frequency of the background deviant was 2% or 4% lower than that of the background standard tones in separate sessions (Fig 3A & 3B). The testing order of the two background deviant saliences was counterbalanced across birds. Similar to the baseline condition, the frequency separation between the target and background stream was 8 STs. Condition 2 differed from condition 1 merely in that the background deviant in condition 2 consistently appeared at the 5^th^ tone in the background stream of both the testing and sham trials (Fig 3C & 3D).

### Data analysis

The thresholds of frequency deviants the birds could discriminate in the target stream were calculated in 3 conditions: without the background deviant in baseline, with sequentially unpredictable background deviants in condition 1, and with sequentially predictable background deviants in condition 2. Sessions with higher than 20% false alarm rates or lower than 70% hit rates were discarded. The last 20 trials for each stimulus were collected to calculate the thresholds for statistical analysis in each condition. The thresholds at d’ = 1.5 were calculated according to the signal detection theory using the hit and false alarm rates in each condition. Analyses were performed in SPSS 24.0. For baseline, a one-way repeated measures ANOVA (3 target deviant positions) was conducted on the thresholds obtained at each target deviant location; in condition 1, a three-way repeated measures ANOVA (3 target deviant positions × 2 background deviant saliences × 3 background deviant positions) was conducted on the thresholds obtained at each combination of target and background deviant sequential positions; in condition 2, a two-way repeated measures ANOVA (3 target deviant positions × 2 background deviant saliences) was conducted on the thresholds obtained at each target deviant position.

## Results

### Baseline experiment

**Fig 4:**
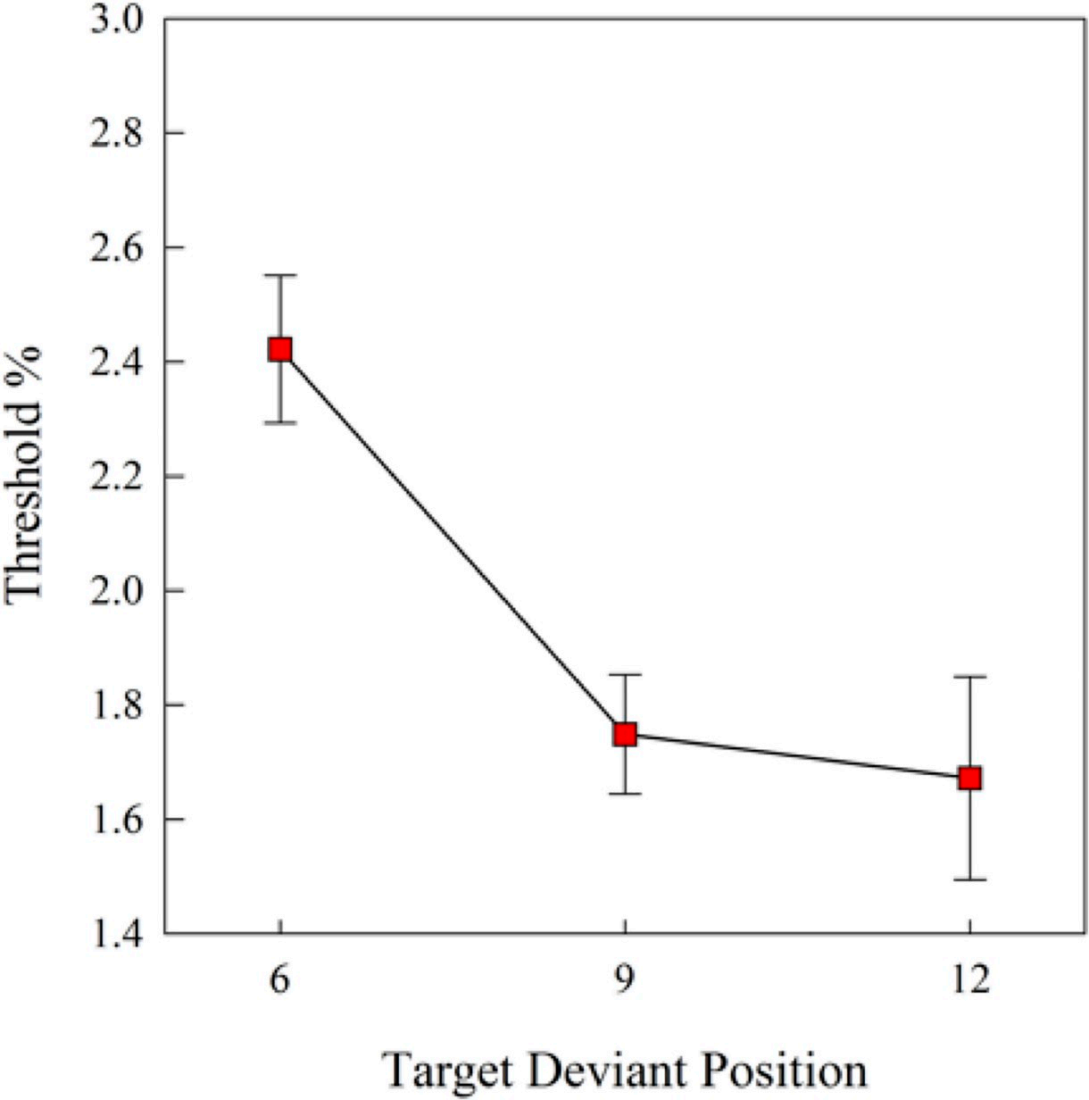
Mean thresholds of frequency deviants as a function of the target deviant positions in the baseline condition (N = 7). Error bars represent SEM.

The one way repeated measures ANOVA (3 target deviant positions) indicated a significant main effect of target deviant positions (*F*(2, 12) = 12.83, *p* = 0.001). Bonferroni post-hoc pairwise comparisons indicated that the thresholds obtained at the 9^th^ (*p* = 0.01) and 12^th^ tones (*p* = 0.04) were significantly smaller than those obtained at the 6^th^ tone, as shown in Fig 4. Hence, congruent with the observation from a subjective auditory streaming paradigm [5], we also observed the buildup effect in birds performing an objective auditory streaming task. Here, as the birds hear the AAAABAB… sequence for a longer time, the birds can discriminate a smaller frequency deviant in the target stream. The buildup effect reaches an asymptote after the 9^th^ tone, evidenced by a nonsignificant difference between the thresholds obtained at the 9^th^ and 12^th^ tones, as shown in Fig 4.

### Condition 1

**Fig 5:**
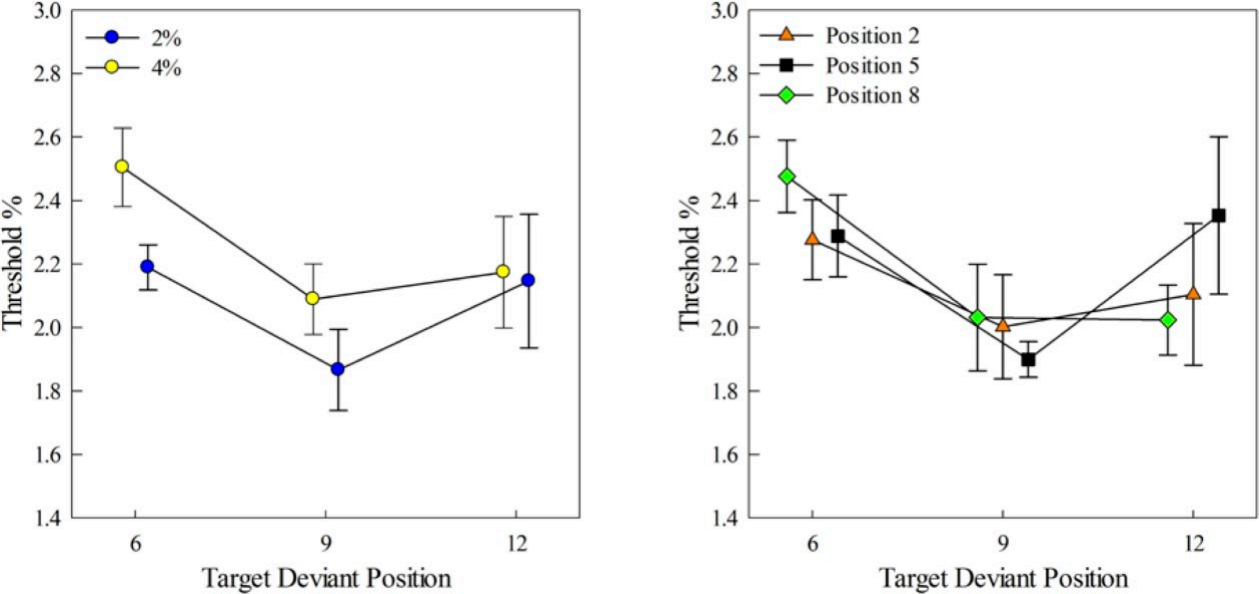
Mean thresholds as a function of the target deviant positions in condition 1. Left: The threshold at each target deviant position was averaged across the three background deviant positions. The thresholds with 2% and 4% background deviant saliences are represented by the blue and yellow circles, respectively (N = 7). Right: Mean thresholds at each target deviant position were averaged across the two background deviant saliences. Each background deviant position is represented by a separate line. Error bars represent SEM.

The three-way repeated measures ANOVA (3 target deviant positions × 2 background deviant saliences × 3 background deviant positions) indicated a significant main effect of background deviant salience (*F*(1, 6) = 27.74, *p* = 0.002). Bonferroni post-hoc pairwise comparisons indicated that a background deviant elicited an involuntary attention switch from the primary task, as evidenced by a higher threshold obtained with the 4% background deviant than that obtained with the 2% background deviant (*p* = 0.002). The mean thresholds obtained with the 4% and 2% background deviants were 2.26%±0.11% (SEM) and 2.07%±0.12% (SEM) respectively (Fig 5 left).

The buildup effect was deteriorated by the unpredictable background deviants. Compared to the baseline condition, where no deviants appeared in the background stream, in condition 1, the main effect of target deviant positions was not significant (*F*(2, 12) = 3.22, *p* = 0.08) (Fig 5 right). The background deviant position factor was also not significant (*F*(2, 12) = 0.40, *p* = 0.68). None of the pairwise interactions between the three factors were significant, nor was the interaction between all three factors (*p* > 0.05). Thus, the only factor that appears to influence attention in condition 1 is the salience of the target deviant.

### Condition 2

**Fig 6:**
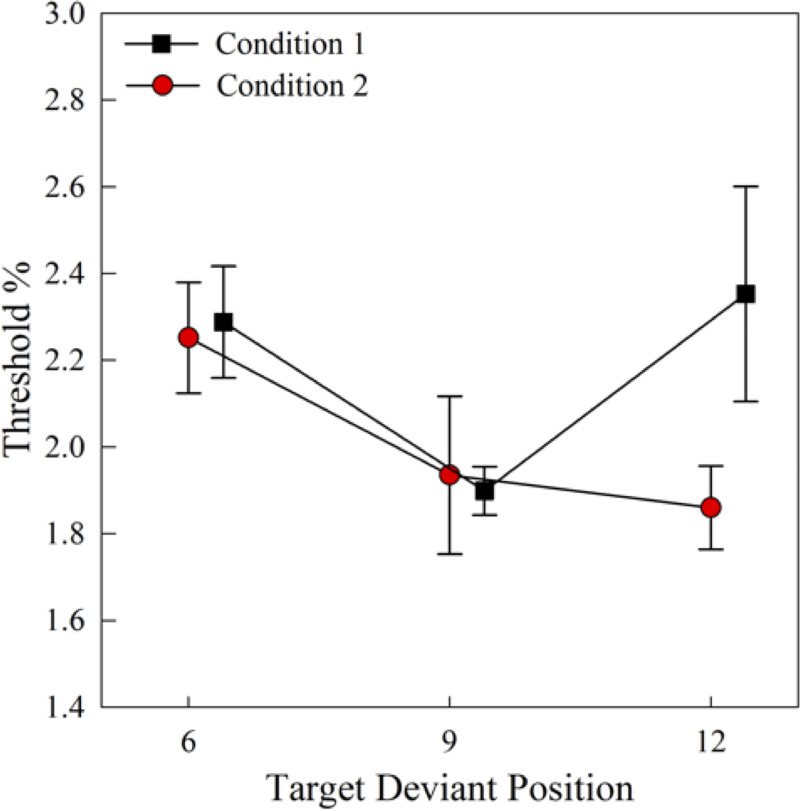
**Mean thresholds for background deviant position 5 as a function of the target deviant positions in conditions 1 and 2 with sequentially unpredictable (black squares) and predictable (red circles) background deviants (N = 7), averaged across the 2 different background deviant saliences in each condition.** Error bars represent SEM.

In condition 2, the two-way repeated measures ANOVA (3 target deviant positions × 2 background deviant saliences) indicated a significant main effect of target deviant position (*F*(2, 12) = 5.07, *p* = 0.03). The main effect of target deviant position in condition 2 was mainly driven by the difference between thresholds obtained at the 6^th^ and 12^th^ tones (*p* = 0.007). The threshold obtained at the 6^th^ tone (2.25%±0.13%) was significantly higher than that obtained at the 12^th^ tone (1.86%±0.1%), as shown in Fig 6. However, a two-way repeated measures ANOVA (3 target deviant positions × 2 background deviant saliences) conducted on the results obtained from the same stimuli in condition 1, where the background deviant appeared at the 5^th^ tone, indicated a significant main effect of background deviant salience (*F*(1, 6) = 8.89, *p* = 0.03), but no significant main effect of target deviant position (*F*(1.17, 7) = 2.80, *p* = 0.14, Greenhouse-Geisser adjustment of sphericity), and no significant interaction (*F*(2, 12) = 0.73, *p* = 0.5).

Unlike in condition 1, where the 4% background deviant distracted attention more than the 2% background deviant, in condition 2 no significant differences were observed between the two different background deviant saliences (*F*(1, 6) = 2.32, *p* = 0.18). Hence, sequentially predictable background deviants tend to mitigate the attention capture effects elicited by different salience levels of background deviants.

## Discussion

Several comparative animal models have been used to investigate the neural mechanisms of auditory attention in the auditory streaming process. For example, knock-out mice that lack the top-down connections to outer hair cells showed poorer performance in response to auditory distractions [68]. Selective attention can facilitate the neuronal plasticity in primary auditory cortex in ferrets [16]. However, few studies have addressed how an awake and behaving animals’ perception in auditory streaming tasks is affected by bottom-up and top-down attention. Here, we trained birds in an objective auditory streaming paradigm, where the traditional ABAB… patterned pure tone sequence was modified to train the birds to selectively attend to the target stream of a sound mixture. Additionally, we added two conditions with a task irrelevant distractor in the background stream to discourage the birds from paying global attention to the whole sound. The sound intensity used here was 90 dB SPL, which was higher than that used in our previous study (70 dB) [5]; this was designed to make the discrimination task easier for the birds. Previous studies in humans and other animal species have indicated that frequency difference limens generally decrease as the stimulus intensity increases [82, 83].

In the baseline condition, we replicated the buildup effect in auditory streaming which was observed previously in a subjective paradigm using the same species [5]. When comparing the buildup process at the same frequency separation (8 STs) between the target and background streams, we observed a faster buildup process in the current study. The buildup effect here reached an asymptote after about 1.92 s, while it took about 6 s to reach asymptote in the subjective paradigm. A plethora of literature has demonstrated that cortical neurons adapt faster when presenting the ABA-… or ABAB… patterned stimuli at a faster repetition rate [22, 24, 28], which leads to a faster streaming process [84]. Therefore, it is likely that the faster repetition rate of sound stimuli used here leads to the accelerated buildup effect. Also, human studies have indicated that attending to the sound facilitates neural selectivity in auditory streaming [38], and that selectively attending to a subset of sounds can attenuate the processing of unattended sounds in the auditory streaming task [85, 86], especially for sounds that generate an ambiguous perception [87]. Compared to the subjective paradigm, where the birds were trained to globally attend to the whole sound stimulus, the objective paradigm encouraged the birds to selectively attend to the target stream embedded in the sound mixture to obtain the best performance. Hence, the faster buildup process observed here could also be ascribed to the birds being trained to selectively attend to the target stream to succeed in the task. Finally, it is likely that the increased stimulus intensity contributed to the faster buildup process. Further studies need to be conducted to address how different stimulus intensities can affect the buildup of the selective adaptation process differently in the auditory streaming tasks.

A number of human studies have investigated attention capture caused by task irrelevant deviants, and how attention capture could be affected by the top-down process [55–59]. Here, we trained the birds to pay selective attention to a target stream in a two-stream sound mixture, in which a sequentially unpredictable or predictable distractor could appear in the task irrelevant background stream. We measured the birds’ sensitivity to a frequency change that randomly appeared in the target stream to gauge the attention capture effect brought by the background distractor. In condition 1, the sequentially unpredictable background deviant generally deteriorated the birds’ sensitivity in the primary task relative to the baseline (no background deviant) task. Additionally, the more salient the background deviant, the greater the deterioration of the performance, as indicated by an increase in the discrimination threshold and a reset of the buildup process, as shown in Fig 5 (left). These results qualitatively match the results in studies on humans, where more salient distractors tend to elicit larger ERPs in an oddball paradigm [60] and worse performance in a dichotic listening task [61].

The buildup process failed to recover over the time course of the stimuli used here. For example, when the background distractor appeared at the 2^nd^ tone, the birds’ thresholds did not improve as the target deviant appeared later in the target stream (triangles in Fig 5, right). Previous dichotic listening studies in humans indicated that the distractor elicited neural response and the behavioral impairment disappeared after 560 ms of the capturer onset [49, 61]. Here, the longest recovery time between a background distractor and the target deviant was 1360 ms (i.e., when the background distractor was at the 2^nd^ background tone and the target deviant was at the 12^th^ target tone). No statistically significant recovery of sensitivity was observed during this time period. The longer recovery time observed in birds relative to humans may be ascribed to three possible reasons. First, in the dichotic listening task, spatial separation between the target and background streams may make it easier for human subjects to reorient attention back to the target stream after the involuntary attention switch. As in [88], a nonspatial attention shift led to a slower behavioral response than a spatial attention shift. However, since the target and background streams were delivered without spatial separations here, it may take longer for the birds to reorient attention back to the target stream for the buildup process to have an effect on perception. Second, in the human studies, no sound was presented during the 560 ms recovery period [57, 60, 61], while here the competing two-stream mixtures continued during the 1360 ms ‘recovery period’, and the birds needed to reorient their attention selectively back to one of the streams in the competing sound mixtures. It has been shown that the deterioration effect of a visual distractor gets exacerbated for tasks requiring higher cognitive load (e.g., working memory or selective attention) [45]. Hence, it is likely that in the auditory modality, the increased cognitive load during the recovery period prolonged the impairment effect caused by attention capture. Although the top-down controlled and bottom-up triggered attention tends to activate segregated cortical areas in vision [1] while triggering overlapping cortical networks in the auditory modality [50]. Lastly, it could also be a species difference between birds and humans in the recovery of the buildup process after attention capture.

In condition 1, where the background distractors were sequentially unpredictable, we observed a trend of a facilitation effect when the background distractor appeared right before the target deviant after several repetitions of the AB- pattern. For a distractor that appeared at the 5^th^ background tone, the threshold obtained at the 9^th^ target tone was smaller than the threshold obtained at the 6^th^ target tone (Fig 5, right, squares); for a distractor that appeared at the 8^th^ background tone, the threshold obtained at the 12^th^ target tone was smaller than the threshold obtained at the 6^th^ tone (Fig 5, right, diamonds). The birds’ false alarm rates did not significantly increase in condition 1 compared to the baseline condition (FA% baseline: 9.24%±1.44%; Condition 1 2%: 7.55%±0.90%; Condition 1 4%: 8.82%±1.20%); for all sham trials the sequentially unpredictable distractor still appeared in the background stream. Hence, it is not the case that the birds paid global attention to the whole sound as a task strategy, with a background distractor that appeared right before a target deviant enhancing the perceptual salience of that target deviant. On the other hand, when the distractor appeared at the 2^nd^ background tone, no differences were found among thresholds obtained at the 6^th^, 9^th^, or the 12^th^ target tones. This indicates that the reset of the buildup effect failed to recover even for the stimulus with the longest recovery period. Also, for distractors that appeared at the 5^th^ background tone, the thresholds obtained at the 12^th^ target deviant were not significantly smaller than those obtained at the 6^th^ tone. Hence, it is less likely that the facilitation effect is caused by the recovery of the buildup process. Instead, it is more likely that either the background distractor elicited a higher short lived arousal level, which facilitated the discrimination of the target deviant right after the background distractor [57], or that the background distractor temporally narrowed the attentional spotlight to the increase in frequency, which enhanced the subsequent discrimination of the target frequency deviant (which was always at higher frequency than target standard). In vision, this temporal attentional spotlight narrowing effect is also short lived, and disappears after 250 ms [66]. This may explain why the sensitivity only increased for target deviants that appeared right after the background distractor, and not the target deviants that appeared later.

In condition 2, we observed that the sequential predictability of background distractors was conducive to the recovery of the buildup process after the attention capture. The birds’ sensitivity to the 12^th^ target deviant was significantly smaller than to the 6^th^ target deviant in condition 2 (Fig 6, circles). However, in condition 1 where the background distractors were sequentially unpredictable, for trials with a background distractor that also appeared at the 5^th^ background tone, no difference was observed between thresholds at the same two target deviant positions (6^th^ and 12^th^) (Fig 6, squares). Studies using the oddball paradigm in humans found that the predictability of the task-irrelevant feature dimension of the distractor can enhance listeners’ performance in the task-relevant feature dimension of the same distractor [63]. In a dichotic listening task, listeners perform better in the primary task when the unattended stream has a predictable sequential sequence than when the unattended stream has an unpredictable random sequence [65]. In a frequency discrimination task in the same species of birds used in the current study, predictability of the location of the target in a tone sequence was found to not hinder acuity [78]. In that same experiment, humans were considerably affected by target location uncertainty. Here, for the first time in behaving animals, by presenting the exact same sound stimuli in either a sequentially predictable or sequentially unpredictable context, we observed the contextual dependence of top-down processes when dealing with attention capture. Nevertheless, the trend of a facilitation effect was not observed in the context of sequentially predictable distractors. In condition 2, the threshold obtained at the 9^th^ tone, which appeared right after the sequentially predictable background distractor, was not significantly smaller than the threshold obtained at the 6^th^ tone. It is possible that the predictable background distractor increased inhibition activities to the distractor [65], which consequently eliminated the distractor-elicited arousal level and distractor-elicited attentional narrowing. This, in turn, eliminated the facilitation effect. Further experiments should be designed to investigate how temporal relationships between the distractor and the target could affect the attention capture process differently under different contexts (e.g., predictable or unpredictable distractors).

Finally, a possible limitation of the present study was the lack of a manipulation of animals’ sex and age in the experiments. In humans, the effects of attention capture on primary tasks are associated with the vigilance of the central nervous system [44], more specifically, the frontal cortex, which is mainly responsible for the maintenance of the attentional spotlight. It has been shown that older people’s frontal lobes are more sensitive to distractors than those in younger listeners [46, 49], therefore they are more vulnerable to the attention capture effect in attentional tasks. Moreover, females have shown increased response times and stronger novelty P3 amplitude of ERPs to unexpected auditory stimuli [89]. Finally, a recent study in domestic horses (*Equus caballus*) has indicated consistent individual variability in response to the attention capture effect [67]. Hence, future studies should be conducted to investigate the effects of sex and age on the attention capture process in auditory streaming in birds.

## Conclusions

Birds experience the buildup effect in an objective auditory streaming paradigm. The effect attention capture has on birds was qualitatively similar to that in humans: with the manipulation of top-down selective attention, task irrelevant background distractors generally impaired bird’s performance, while some facilitation effect was observed depending on the temporal relationship between the background distractor and the target deviant. Additionally, sequential predictability of distractors can enhance the recovery process of the buildup effect after attention capture. This paradigm can be modified for further studies on the neural mechanisms of goal-oriented selective attention and stimulus-driven attention capture processes in behaving animals.

## Supporting information

S1 File The raw experimental results for individuals across all conditions. (XLSX)
